# A Framework for Multiple Ground Target Finding and Inspection Using a Multirotor UAS †

**DOI:** 10.3390/s20010272

**Published:** 2020-01-03

**Authors:** Ajmal Hinas, Roshan Ragel, Jonathan Roberts, Felipe Gonzalez

**Affiliations:** 1 Robotics and Autonomous Systems, Queensland University of Technology (QUT), Brisbane City QLD 4000, Australia; Jonathan.roberts@qut.edu.au (J.R.); felipe.gonzalez@qut.edu.au (F.G.); 2 Department of Computer Engineering, University of Peradeniya (UOP), Peradeniya 20400, Sri Lanka; roshanr@pdn.ac.lk

**Keywords:** unmanned aerial vehicle, unmanned aerial system, vision-based navigation, search and rescue, vision and action, OODA, inspection, target detection, remote sensing

## Abstract

Small unmanned aerial systems (UASs) now have advanced waypoint-based navigation capabilities, which enable them to collect surveillance, wildlife ecology and air quality data in new ways. The ability to remotely sense and find a set of targets and descend and hover close to each target for an action is desirable in many applications, including inspection, search and rescue and spot spraying in agriculture. This paper proposes a robust framework for vision-based ground target finding and action using the high-level decision-making approach of Observe, Orient, Decide and Act (OODA). The proposed framework was implemented as a modular software system using the robotic operating system (ROS). The framework can be effectively deployed in different applications where single or multiple target detection and action is needed. The accuracy and precision of camera-based target position estimation from a low-cost UAS is not adequate for the task due to errors and uncertainties in low-cost sensors, sensor drift and target detection errors. External disturbances such as wind also pose further challenges. The implemented framework was tested using two different test cases. Overall, the results show that the proposed framework is robust to localization and target detection errors and able to perform the task.

## 1. Introduction

Small, unmanned aerial systems (UASs) now have advanced waypoint-based navigation capabilities, which enable them to collect surveillance [1], wildlife ecology [2] and air quality data [3] in new ways. One capability that is not available in most off-the-shelf UASs is the ability for a higher level of onboard decision-making for vision and action.

The ability to remotely sense and find a set of targets, and descend and hover close to each target is desirable in many applications, including spot spraying in agriculture (such as pesticide spraying for brown planthopper (BPH) [4] and spear thistle weed [5]), inspection and search and rescue operations in a larger area with limited human resources, or in difficult terrain, such as searching for survivors or an aviation black box.

**Figure 1:**
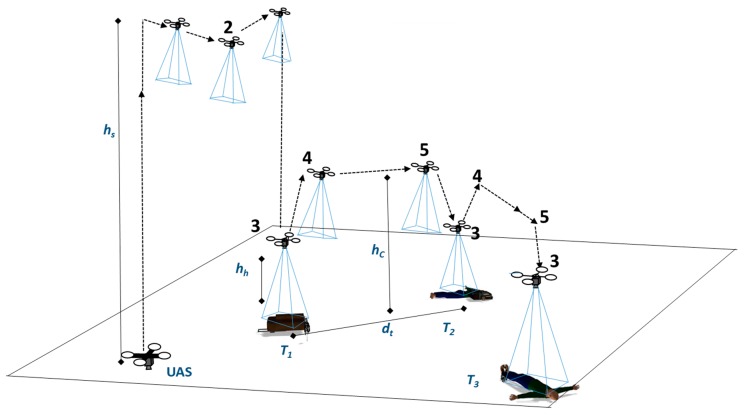
Illustrative search for survivors scenario with multiple objects scattered on the ground. The numbers 1–5 indicate the steps performed in the mission. Steps 3–5 are repeated for each target.

Figure 1 is an illustrative example of a search scenario for survivors. The task can be divided into multiple steps. Initially, the UAS searches for ground targets at search height *h_s_* (1). If target/s are found, the UAS picks a target, based on a criterion such as first detected target and changes its original path and moves toward the target and descends (2). The UAS then hovers above the target closely (*h_h_* = 0.5–2.5) to inspect the target (3). After inspection, the UAS climbs slightly (<2 m) and reaches height *h_c_* and moves laterally to the next target (4). After reaching the next target, the UAS descends (5) and inspects that target (3). The UAS repeats steps 3, 4 and 5 with each target.

Very accurate target detection is needed to perform this task. However, target detection algorithms have a certain degree of uncertainty in real outdoor environments. Typical single frequency GPS receivers have an accuracy of 5–6 m [6,7]. Another challenge is that a greater localization accuracy needed to act on targets. In this paper, our aim is to develop and demonstrate a framework for autonomous vision-based ground target finding and action using a multirotor UAS that is robust to the (i) errors and uncertainties in the localization of the UAS; (ii) errors and uncertainties in localization of the target; (iii) target detection errors and; (iv) the effects of external disturbances such as wind.

The framework uses the high-level decision-making approach of observe, orient, decide and act (OODA) [8,9,10] and a series of techniques to perform the necessary navigation and control and target tracking. The proposed framework was implemented as a modular software system and demonstrated in two different test cases.

The contributions of the paper are summarized as follows: (1) An onboard framework for finding and acting on multiple ground targets which is robust to errors in localization of the UAS, localization of the targets and target detection, and external disturbances; (2) A computationally efficient voting-based scheme to avoid false detections in vision-assisted navigation or target tracking using UASs.

## 2. Related Work

The development of the multiple target detection and action framework is closely related to previous work on vision-based landing, target detection, target tracking, infrastructure inspection and OODA.

The vision-based landing task of UASs is a well-explored area of research. In this task, the UAS guides and descends towards a detected landing pad using vision. Mostly, a single, known target is used to detect the landing area such as a landing pad, and the accuracy of a few meters from the landing pad is usually acceptable. Specialized patterns, such as “H”, are common to improve detection [4,5,6] or control the UAS [11]. Techniques such as Image-Based Visual Servoing (IBVS) and the relative distance-based approaches [7] are common. However, these techniques have limitations for multiple targets when the targets have unknown shapes and sizes or are similar.

Zarudzki et al. [12] explored multiple target tracking using an IBVS scheme and different colors for the targets to separate them. However, the IBVS schemes are limited when all or a subset of the targets are visually similar.

Many researchers have explored the tracking of a moving target [8,9,10,11]. For example, Greatwood et al. [13] used the relative location of an unmanned ground vehicle (UGV) to track it. However, relative location-based approaches are limited for multiple similar targets. In another study, Vanegas et al. [14] demonstrated finding a UGV and tracking it in GPS-denied and cluttered environments using a Partially Observable Markov Decision Process (POMDP) formulation and known obstacle position to correct the UAS pose. However, such knowledge is not available in our work. Tracking of a user-selected object was also demonstrated by Cheng et al. [14]. The authors used a UAS fixed with a gimbaled camera and a Kernelized Correlation Filter (KCF)-based visual tracking scheme to track the selected object. All of these works are limited to a single known target.

Vision-based or assisted navigation of UASs is also a well-studied area of research [15,16,17]. Stefas et al. [18] studied the navigation of a UAS inside an apple orchard. Missions following water channels [19] and roads [20] have also been explored. Other inspection tasks using UASs are power line inspection [21,22] and railway track inspection [23]. In these studies, a common approach is to follow the continuous image feature lines present in the observed structures. However, this research investigates the problem of finding and acting on multiple discrete targets.

In other works for railway semaphore inspection [24] and pole inspection [25], visual control techniques have been explored to fly the UAS around a pole-like structure. However, finding and approaching the target is not considered in these studies.

Vision-based ground target finding and action systems have been studied for a single target [5,26,27]. Multiple ground target finding and action capability have also been demonstrated [28,29]. However, those works did not consider the uncertainties in target detection.

Most previous studies used a control system approach to control the UAS. Another possible approach is a cognitive model-based approach of OODA [30,31]. For example, Karim and Heinze [32] implemented and compared two distinct autonomous UAS controllers. They found OODA is more intuitive and extensible than the control system approach and allows asynchronous processing of information between stages. The work of Karim and Heinze was limited to a simple mission of choosing an alternative waypoint. Priorities in developing a framework include design intuitiveness, extendability, and scalabilities. In our framework, the OODA approach is used to control the UAS.

UASs have been explored for search and rescue (SAR) missions in other related work [33,34,35]. Hoai and Phuong [36] studied anomaly color detection algorithms on UAS images for SAR work. Niedzielski et al. [37] conducted SAR field experiments to guide ground searchers in finding a lost person. In these studies, the focus was limited to identifying the targets from UAS images, and the computation was primarily performed at an offboard computation infrastructure. Some researchers have also documented that the communication delay between the UAS and the offboard computation infrastructure is a severe limitation. However, our focus is to develop an autonomous system that can perform onboard detection in real time and closely approach the detected target to perform a close inspection for more informed decision-making.

The assessment of autonomy level is an important aspect to compare and evaluate the performance of the system presented in this paper. Several models have been proposed to assess the autonomy level of the systems, including autonomy level evaluation [38] formulated by the National Aeronautics and Space Administration (NASA), the Autonomous Control Levels (ACL) method [39] proposed by the U.S. military, and the Autonomy Levels for Unmanned Systems (ALFUS) framework [40] by the National Institute of Standards and Technology (NIST)—each model has its drawbacks [41]. The autonomy level evaluation formulated by NASA is used in this research as it is simple and easy to use for comparison of unmanned systems platforms, regardless of their operating environment.

In summary, the task of vision-based multiple ground target finding and action involves solving problems in detection, tracking, navigation, and action on a set of discrete ground targets where visual uniqueness of the targets cannot always be guaranteed, and targets may also move out of the camera’s field of view (FOV).

## 3. Framework

**Figure 2:**
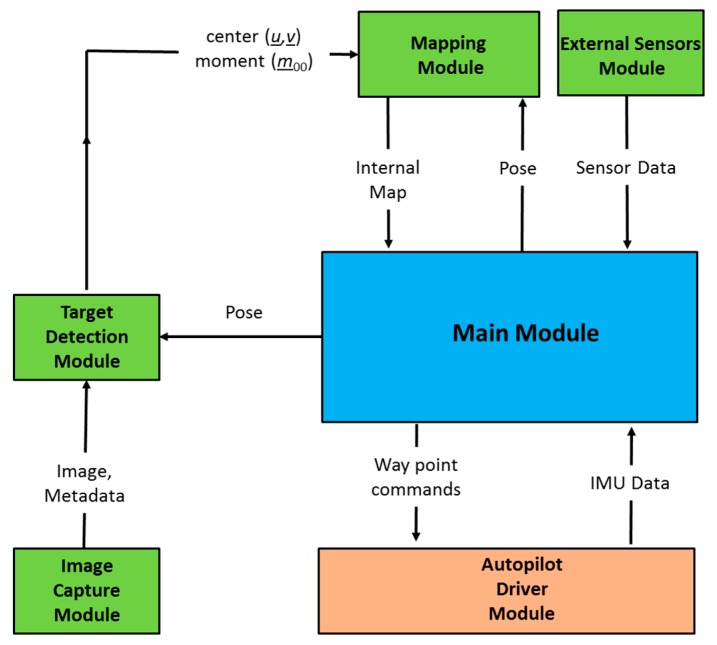
Software system architecture.

The main contribution of this paper is a navigation framework. This framework was developed and implemented as a modular software system. The current implementation of the framework has six modules: main module, image capture module, a target detection module, mapping module, autopilot driver module and external sensor module. Figure 2 describes the software system architecture.

The image capture module (Section 3.1), target detection module (Section 3.2), mapping module (Section 3.3), and the main module (Section 3.5) were developed in C++, and Mavros [42] is used as the autopilot driver module. It is available as a ROS [43] package. The main module performs the orient and decide functions of the OODA loop. The following subsections describe each module.

### 3.1. Image Capture Module

The image capture module captures images from the attached camera sensor and sends the captured images and image metadata, such as timestamp and resolution, to the target detection module.

### 3.2. Target Detection Module

**Figure 3:**
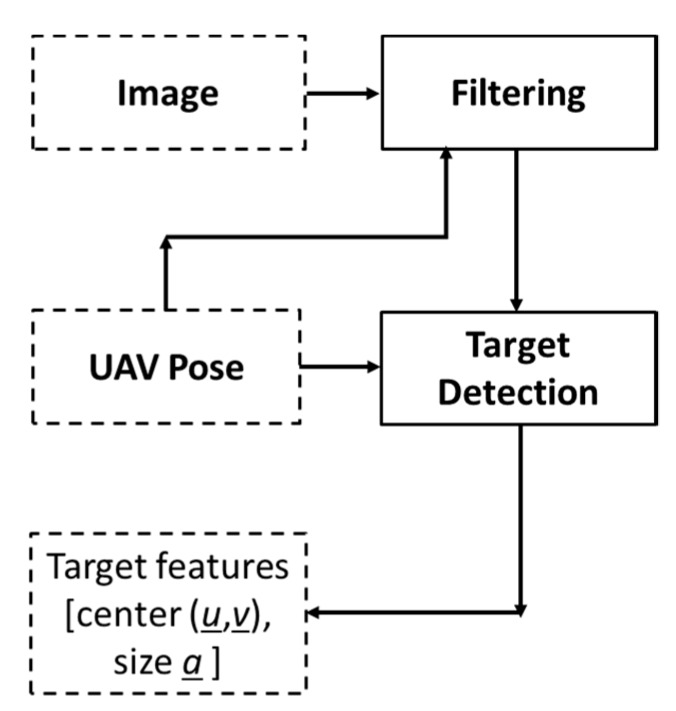
Target detection module.

The target detection module (Figure 3) detects targets and extracts interesting features of the target, such as the center and size, and passes the information to the mapping module. In a ground target finding and action mission, the UAS flies through different sets of altitudes depending on which step (Figure 1) the UAS performs. A static target detection algorithm may not perform well for all altitudes. Therefore, the target detection module receives the UAS pose information from the main module and adjusts the detection method according to the altitude of the UAS for more accurate detection.

The target detection module performs two different functions: filtering and target detection. The filtering function rejects unsuitable images from the processing pipeline and helps to reduce some processing cycles of the onboard computer. The target detection error increases with the rotation rate of the UAS. Therefore, the criterion given in Equation (1) is used to discard the images.

(1) $$\exists n\in Z^{+},\;|\phi_{n}-\phi_{n-1}\left|>\alpha \vee |\theta_{n}-\theta_{n-1}\right|>\alpha \vee |\psi_{n}-\psi_{n-1}|>\alpha$$

Here,$\phi_{n}$, $\theta_{n}$ and $\psi_{n}$ are the roll, pitch and yaw angles of the UAS in the nth frame, and α is the threshold value determined experimentally. UAS images might also be susceptible to motion blur depending on the camera and UAS speed. Therefore, the filtering function can be implemented to include automatic blur detection algorithms described in [44] to filter those images, which is beneficial when more complex target detection algorithms are used. However, our current implementation did not use these methods, considering the processing overhead.

### 3.3. Mapping Module

**Figure 4:**
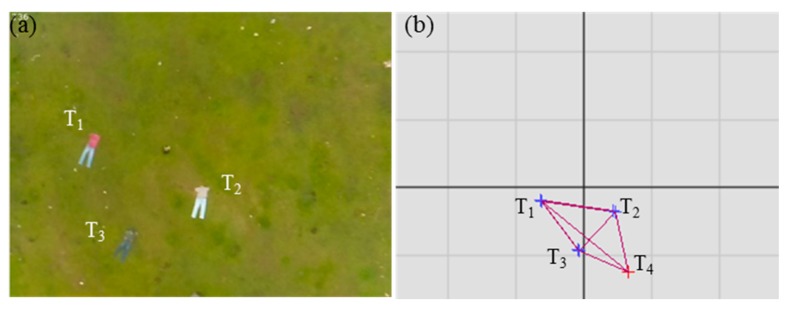
Image with targets (**a**) and corresponding internal map in graphical illustration (**b**).

The mapping module creates an internal map of all detected targets. The map helps to track the targets when they go outside the FOV. The map is also used to compensate for the effects of localization errors due to GPS noise and drift. Figure 4 shows an image (Figure 4a) and the corresponding internal map (Figure 4b) in a graphical illustration. In Figure 4a, the fourth target *T*_4_ is outside the FOV.

The mapping module creates an internal map through the following steps:

#### 3.3.1. Estimate Target Position

The estimate target position step estimates the 3D position of the target in the inertial frame from the outputs of the target detection module. Our previous paper [27] describes the detailed steps of the method.

#### 3.3.2. Track Targets

The track targets step matches the target between each image frame. The estimated position of each target may vary throughout the mission due to inaccuracies and sensor drift. However, the variation between adjacent frames is small; therefore, targets are matched using the Euclidean distance between the estimated target positions in adjacent frames.

Target $T_{i,n}$ and target $T_{j,n-1}$ are the same target if:

(2) $$\exists n,i,j\in Z^{+},\sqrt{{\left({\hat{x}}_{i,n}-{\hat{x}}_{j,n-1}\right)}^{2}+{\left({\hat{y}}_{i,n}-{\hat{y}}_{j,n-1}\right)}^{2}}<d$$

where $T_{i,n}$ is the ith detected target from the nth frame, $\left({\hat{x}}_{i,n},\;{\hat{y}}_{i,n}\right)$ is the position estimation of the ith detected target from the nth image frame, and d is the gating distance.

The filtering stage keeps d within a reasonable limit.

#### 3.3.3. Build Internal Map of Adjacent Targets

When the UAS descends towards a selected target for an action, other targets go out of the camera FOV. However, the target selected for the action can be visible most of the time. An internal map of the targets is built using weighted graph representation to use this property. This map also tracks the locations of the targets already registered, but is undetected in specific frames (false negatives).

The target $T_{i,m}$ and the target $T_{j,n}$ are adjacent if:

(3) $$\exists \;m,n,i,j\in Z^{+},m=n\wedge i\neq j$$

**Figure 5:**
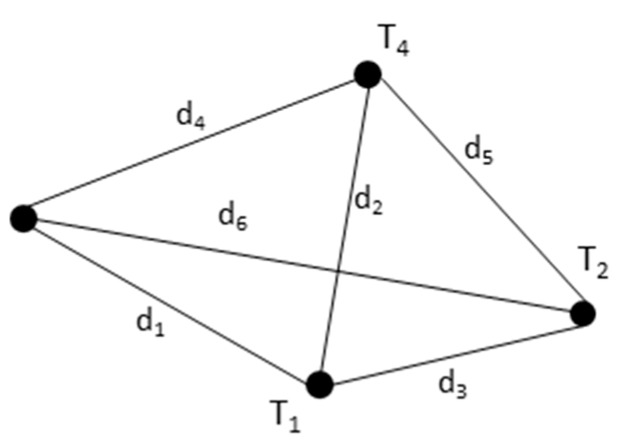
An example of the internal map using four targets.

Figure 5 shows an example of an internal map using four targets. Nodes *T*_1_–*T*_4_ represent the targets, and the weights *d*_1_–*d*_6_ represent the distance between them.

#### 3.3.4. Remove Duplicates

Due to the errors in estimating the target’s position, duplicate nodes (targets) are introduced in the internal mapping step. This step removes these duplicate nodes and their vertices from the map. The node $T_{i}$ and its vertices are removed if:

(4) $$\exists n,i,j\in Z^{+},\sqrt{{\left({\hat{x}}_{i,m}-{\hat{x}}_{j,n}\right)}^{2}+{\left({\hat{y}}_{i,m}-{\hat{y}}_{j,n}\right)}^{2}}<d$$

#### 3.3.5. Update Position

Due to detection errors in the target detection module, an already registered target may not be detected in adjacent frames, or the target may go outside the camera FOV when the UAS is flying at a lower altitude. In this case, the positions of the previously registered but not visible (not detected/out of FOV) targets are updated in this step by considering one of the visible targets as a base target. The updated location of the other target is given by:

$$\exists \;n,i,j,k\in Z^{+},\;i\neq k$$

(5) $${\hat{x}}_{i,n}={\hat{x}}_{k,n}+D{\hat{x}}_{k,j}$$

(6) $${\hat{y}}_{i,n}={\hat{y}}_{k,n}+D{\hat{y}}_{k,j}$$

Here the *k*th target is a base target.$D{\hat{x}}_{k,j},\;D{\hat{y}}_{k,j}$ are the distance between the *k*th and *i*th targets along the *x* and *y* axis, respectively. Here the distances are estimated using the *j*th frame.

#### 3.3.6. Remove False Targets

Very accurate target detection is needed for reliable navigation of the UAS because false positives and false negatives may change the UAS’s path and make the entire operation inefficient. All target detection algorithms have a certain degree of uncertainty unless the target is very simple. The degree of uncertainty may increase with the distance between the target and the UAS. In an outdoor environment, other factors such as fog, time of day, and lighting changes may also increase this uncertainty.

It is found that false positives are usually not persistent across all images. A weighted voting scheme is used to reject false positives. In this scheme, each target $T_{i}$ holds votes $V_{i}$. If the target $T_{i}$ is detected in a frame, it casts $w_{d}$ number of votes:

(7) $$\exists n,i,j\in Z^{+},\;V_{i}\leftarrow V_{i}+w_{d}$$

where $w_{d}$ can be a simple constant value or modeled as a complex function of other parameters such as distance, weather, time of day and illumination conditions, which determine the certainty of detection. For example, detection at a lower altitude may be assigned more votes than detection from a higher altitude. The optimal function of $w_{d}$ is specific to each particular application.

If the target $T_{i}$ is not detected in a frame but its location is inside the FOV, it casts $-w_{n}$ number of votes (Equation (8)). The value of $w_{n}$ can also be determined similar to $w_{d}$:

(8) $$\exists \;i,w_{n}\in Z^{+},\;V_{i}\leftarrow V_{i}-w_{n}$$

The target $T_{i}$ is removed if:

(9) $$\exists \;i\in Z^{+},\;V_{i}<G$$

The value of the G is experimentally determined.

### 3.4. External Sensors Module

The external sensor module allows the connection of sensors such as sonar directly to the onboard computer for additional state estimation information. In our implementation, only an ultrasonic module is used as an external sensor to compensate for altitude measurement errors induced by strong gusts in barometric height measurement used in typical low cost autopilots.

### 3.5. Main Module

**Figure 6:**
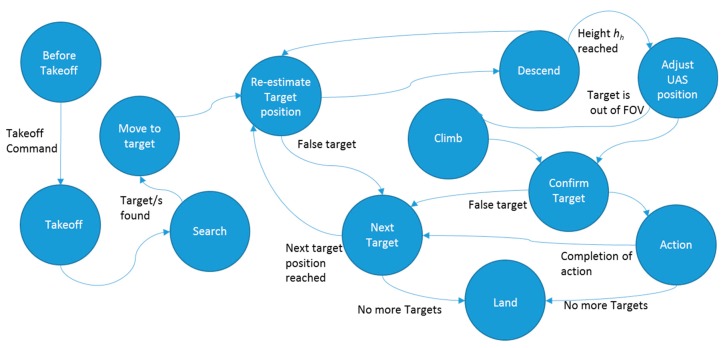
The finite state machine (FSM) of the main module.

A finite state machine (FSM) model is used to implement the main module of the system. Figure 6 shows the finite state machine. The state of the system is controlled by following an OODA loop. Here, a state represents the current belief of the system. According to the observations received from other modules, the system orients and transitions to the next suitable state.

If a target or targets are observed in the search state, the system is updated to the *move to target* state. In the *re-estimate target position* state, the UAS moves laterally towards the target and updates the target position. In the *descend* state, the UAS descends by a predefined amount of height. In the *adjust* state, the UAS aligns its *x*, *y* position above the target by the proportional controller defined by Equations (10) and (11). This controller also assists the UAS to maintain its hovering position against external disturbances such as wind.

(10) $$x_{n}=x_{c}+(U_{o}-u)K/R_{u}$$

(11) $$y_{n}=y_{c}+(V_{o}-v)K/R_{v}$$

In these equations, ($x_{n}$, $y_{n}$) is the output position of the controller, ($x_{c}$, $y_{c}$) is the current UAS position and $\left(U_{o},\;V_{o}\right)$ is the optical center of the camera sensor in pixels. (u, v) is the center of the target in pixels, K is proportional gain, and $R_{u}$ and $R_{v}$ are the horizontal and vertical resolutions of the image.

In the *climb* state, the UAS increases its height by a small amount (<2 m) and transitions into the *confirm target* state. The *confirm target* state confirms the availability of the target. If the availability of the target is not confirmed, it will be removed from the internal map, considering it as a false positive. Actions such as inspection or spraying are performed in the *action* state. After completing the action, target $T_{i}$ is selected as the next target if:

(12) $$\exists \;i,j,l\in Z^{+},(D_{i}=\min_{j=\left\{1\ldots l\right\}}D_{j})\wedge V_{i}>H$$

where $D_{i}$ is the distance to the target $T_{i}$ and $V_{i}$ is the number of votes held by the target $T_{i}$. H is the minimum number of votes that qualify the target as a valid target for a visit. If there is no target to satisfy the criterion (12), the UAS lands at a predefined position.

### 3.6. Autopilot Driver Module

The autopilot driver module acts as an intermediary between the autopilot firmware and the onboard computer system. This module provides data such as autopilot state, inbuilt sensor data and battery state to the main module. Commands from the main module are also translated into autopilot specific control commands.

## 4. Experiments

### 4.1. Hardware System

**Figure 7:**
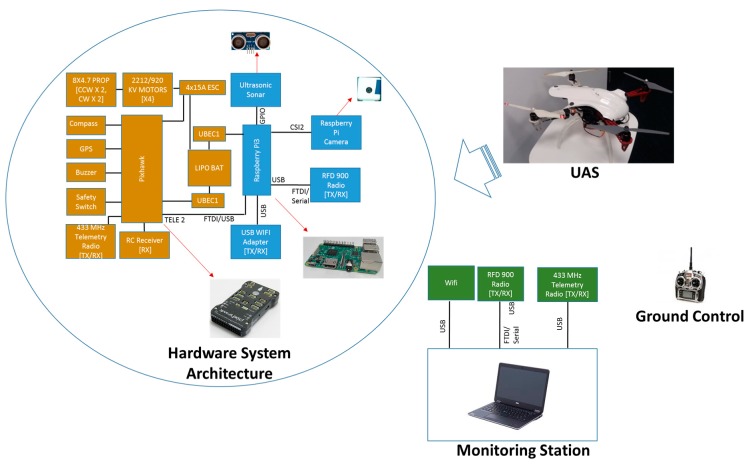
Hardware system used in the experiments.

A quadrotor UAS was developed to conduct outdoor field experiments using a Pixhawk 2 open source autopilot and a DJI F450 frame. The Pixhawk consists of accelerometers, gyroscopes, barometer sensors and other devices on a single board. Figure 7 shows the hardware system used in the experiments. The hardware system architecture shows the components integrated into the UAS and their interconnection, with images of important components. The Raspberry Pi3 onboard computer, camera and sonar are attached under the UAS.

The implemented framework was fully executed in the onboard Raspberry Pi3 computer. A ground control RC transmitter was used only to trigger the system.

### 4.2. Field Experiments

As mentioned already, our focus is to develop and demonstrate a framework for vision-based multiple ground target finding and action using a multirotor UAS that is robust to the (i) errors and uncertainties in localization of the UAS; (ii) errors and uncertainties in localization of the target; (iii) target detection errors; and (iv) effects of external disturbances such as wind. In this paper, we have validated our framework using two different test cases: (1) Finding and inspection of various type of objects scattered on the ground in search and rescue after a plane crash. (2) Finding and inspection of multiple red color ground objects.

**Figure 8:**
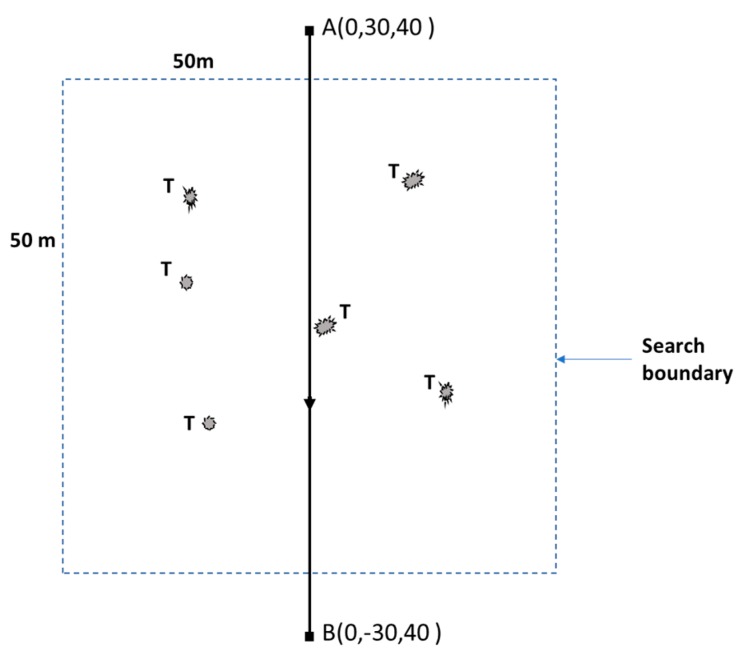
Test case scenario. The dotted square (50 m × 50 m) indicates the search boundary. T indicates scattered targets within the search boundary. Arbitrary shapes of the targets are used to indicate that the targets are not defined by their shape. Waypoints are indicated by (•).

Figure 8 shows the scenario used for both test cases. The UAS has to take off to the search height *h_s_* (e.g., 40 m) and fly from the waypoint *A* to the waypoint *B* and search for targets. If any target/s are detected, the UAS picks a target and changes its flight path, descends and hovers above each target autonomously, as shown in Figure 1, steps 3, 4 and 5. Six targets were scattered randomly. The search boundary was 50 m × 50 m. The test was repeated multiple times, and the exact positions of the targets were randomly changed for different tests. However, a minimum separation distance of 4 m was kept between the targets, and the visibility of all targets in the FOV of the camera at search height *h_s_* (40 m) was maintained. Arbitrary shapes in different colors indicates the targets in Figure 8.

The experiments presented here used several parameters related to the framework and the hardware system used in the experiments. Table 1 lists these parameter values.

Parameters camera resolution and frame rate describe the camera used. Other parameters, such as threshold rotation rate, gating distance, proportional gain, votes for a detection, votes for a non-detection, removal cut-off and valid target cut-off are related to the framework and determined using simulation and trial-and-error experiments.

These framework-related parameters have impact on the performance of the framework. For example, the gating distance *d* was set to 2 m in our experiments. Setting a larger value may label two different targets as the same target while setting a smaller value may label the same target in adjacent frames as different targets. The valid target cut-off *H* was set as 5. Therefore, if a target holds less than five votes, it will not be considered as a possible candidate for a visit or inspection. Hence, a target that appeared in less than five images will be ignored by the UAS. Reducing this value may increase the chance of false targets to become a valid target. Similar effects are possible with the value for removal cut-off *G.* These values can be adjusted depending on the accuracy of the target detection method used. For an example, a lower value of valid target cut-off *H* is adequate for more accurate target detection algorithm.

#### 4.2.1. Test Case 1: Finding and Inspection of Objects Scattered on the Ground in Search and Rescue after a Plane Crash

**Figure 9:**
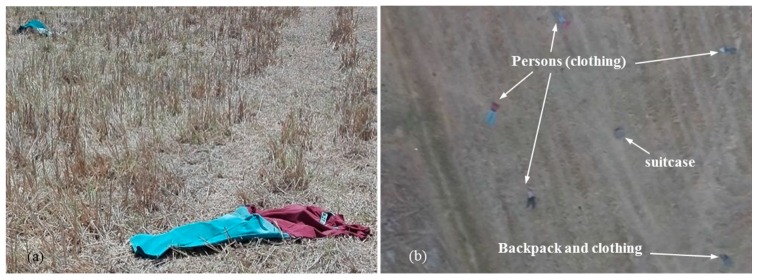
(**a**) Objects scattered at the experimental site in the test scenario; (**b**) Top view of the experimental site, and target objects from a UAS image.

In this test case, a scenario was formulated to represent objects scattered in a search and rescue mission after a plane crash. The unmanned aerial system (UAS) must autonomously find these targets and conduct a close inspection at a low altitude. This capability is very useful in search and rescue operations in a larger area with limited human resources or in difficult terrains, such as searching for survivors or a plane’s black box. Figure 9a shows objects scattered at the experimental site from the ground view. Figure 9b shows the test site and targets from a UAS image. Images from Malaysia Airlines flight MH17 crash site [45,46,47] were used as references. Multiple objects such as clothing to represent people, and suitcases and backpacks were used as targets. In this test case, the targets have different shapes, colors and sizes, and the possibility of false detection is relatively high because of the generalized target detection algorithm.

**Figure 10:**
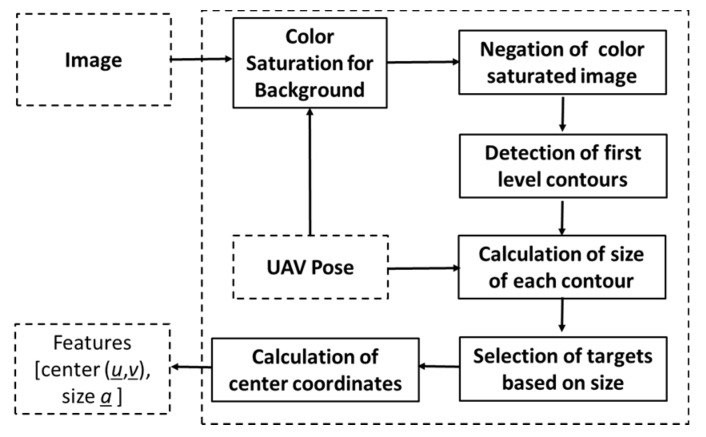
Implementation of the target detection function in the search and rescue test case.

In order to detect various types of objects present in a vegetated area, the target detection algorithm illustrated in Figure 10 was implemented in the target detection function of the target detection module (see Figure 3).

In the first step, the image is saturated for the background color (e.g., green). In the second step, the resulting binary image is negated. In the third step, first level contours are detected. Next, the approximate size a (area on the ground) of each contour is calculated using Equation (13).

(13) $$a=m_{00}\;\frac{z^{2}}{f^{2}}$$

Here $m_{00}$ is the 0th moment, z is the altitude of the UAS, and f is the focal length of the camera. In a binary image, 0th moment $m_{00}$ is defined by:

(14) $$m_{00}=\sum_{u=0}^{u=w}\sum_{v=0}^{v=h}J\left(u,v\right)$$

where w and *h* are the width and height of the blob, respectively. J(u, v) is the value of the pixel at (u, v).

In the next step, the targets were selected based on size. Very small and large targets were omitted. The size of targets must be configured according to the specific application. The primary step of the detection algorithm is color saturation for the background, such as green vegetation. A simple detection algorithm was selected in order to show the capability of the framework in the presence of a high number of false positives.

Test case 1 was repeated five times. Table 2 summarizes the results. The UAS visited all six targets in three of the five flight tests. One target was missed in two tests because the number of votes acquired by the target was less than the minimum number of votes H to qualify to visit the target. An analysis of experimental data also showed that in three tests, the system (main module) did not activate the action state and considered one of the targets as a false detection. A detailed investigation revealed that this was due to detection failure at low altitude (2–4 m). A possible reason might be the auto adjustment settings of the Raspberry Pi camera used.

**Figure 11:**
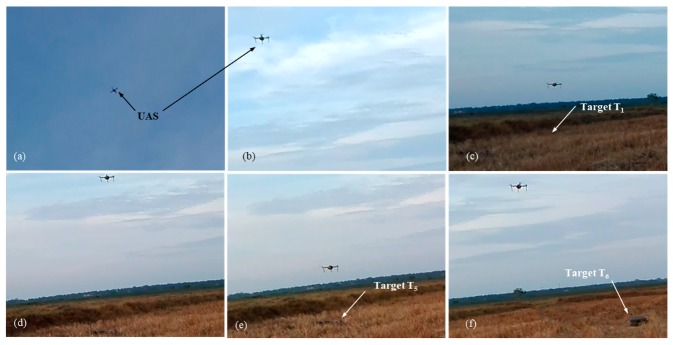
Pictures from test case 1-test 5: (**a**) UAS searches ground target; (**b**) UAS descends towards target *T*_1_; (**c**) UAS inspects target *T*_1_; (**d**) UAS moves towards target *T*_5_; (**e**) UAS inspects target *T*_5_; (**f**) UAS moves towards target *T*_6_.

Figure 11 shows the selected images from test 5.

**Figure 12:**
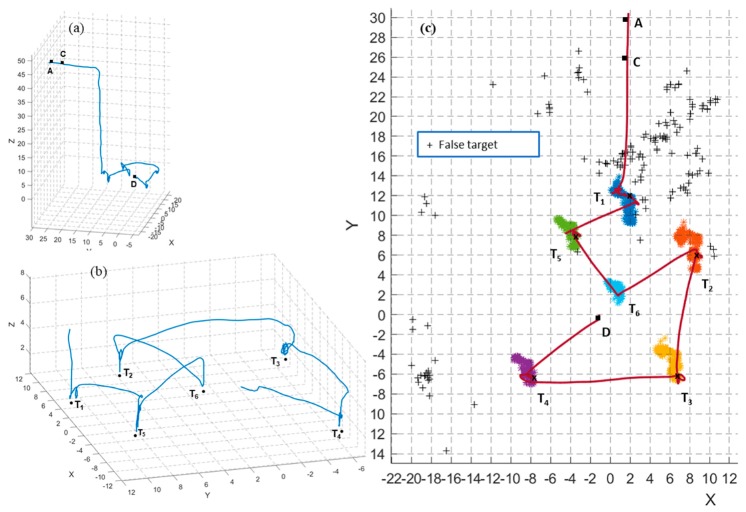
Results from flight test 5: (**a**,**b**) 3D view of the flight trajectory in full and zoomed view of the visiting targets and action part; (**c**) Top view of the flight trajectory and scatter plot of estimated target positions in the full mission of flight test 5. (**+**) indicates false targets. (**x**) indicates the estimated position of the target when the UAS was performing the inspection (hovering above the target). Waypoint B is omitted to increase the clarity of the plots.

Figure 12 shows the results from test 5. In Figure 12a, the UAS searches for the targets while flying from waypoint A to B. At point C, the first target is recognized, and the finite state machine state changes to *move to target* state. The UAS moves towards the first target designated *T*_1_ (step 2, Figure 1) and performs an inspection (hovering above the target for 3 s). Next, the UAS climbs to the cruising height *h_c_* (4 m) and moves to the next nearest adjacent target *T*_5_. The process repeats until the UAS has visited all six identified targets.

However, in this test, the action state is not activated for target *T*_6_. The target detection module failed to detect target *T*_6_ in the confirm state of the finite state machine. *T*_6_ was considered a false target, and the UAS moved to the next target *T*_2_. This shows the autonomous re-planning ability of the system without getting stuck with target *T*_6_.

Figure 12c illustrates the top view of the flight trajectory and the scatter plot of the estimated target positions throughout the full mission. The plot shows several false targets. Moreover, ideally, the position of true targets *T*_1_–*T*_6_ must appear as points. However, they appear like a cloud due to the variation in the estimates because of the localization errors. The highest variation calculated as the furthest distance between two estimates is 5 m in *T*_2_. In total, 117 different possible targets were detected throughout the mission, but only six of them—the actual targets—were visited by the UAS. Therefore, the developed framework can overcome localization errors and target detection errors in target finding and action tasks.

#### 4.2.2. Test Case 2: Finding and Inspection of Multiple Red Color Ground Objects

**Figure 13:**
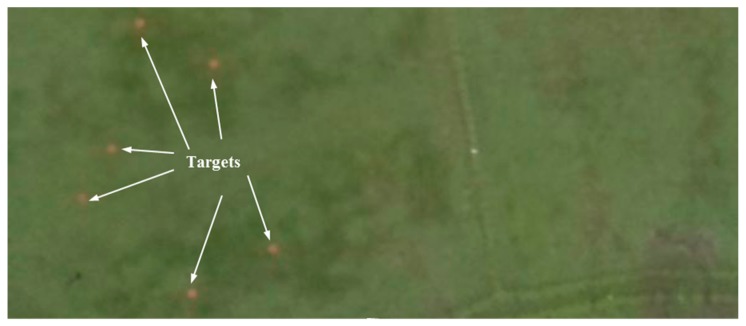
Top view of the experimental site and target objects from a UAS image.

**Figure 14:**
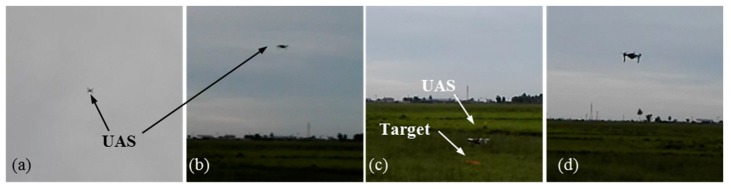
Pictures from a test in test case 2: (**a**) UAS searches ground targets; (**b**) UAS descends towards targets; (**c**) UAS inspects a target; (**d**) UAS moves towards another target for inspection.

In this test case, the UAS must fly according to the same scenario described in Figure 8 and find multiple red color ground objects and approach them and conduct a close inspection at a low altitude. Six similar red color circles with 0.2 m radius were used as targets. For this test case, only the target detection function of the target detection module was modified. Instead of the detection algorithm described in Figure 10, a simple red color detection algorithm was implemented. Similar targets were used in order to demonstrate that the framework’s capability, even in the absence of additional information such as unique color and shape for tracking and control. Figure 13 shows the test site and targets from a UAS image. Figure 14 shows the selected images from one of the tests. Test case repeated five times as before. UAS has visited and inspected all six targets in all five tests. A link to the video of the test is given at the end of the paper. Due to the similarity to the test case 1 results, a detailed discussion of the results is avoided.

The test case 2 was conducted under heavy rain cloud. However, all the tests were successful is implicates the robustness of the framework to such conditions as well.

## 5. Discussion

To our knowledge, no previous work has attempted multiple ground target finding and action using UAS. Related work on vision-based landing provides some insights. However, the techniques used in those studies are mostly limited to a single target and are only demonstrated at low altitudes (<5 m) where the effects of detection and localization errors have minimal impact. One possible technique for multiple target finding and action is to estimate the targets’ positions from the image and guide the UAS towards each target. However, this technique requires a more precise and accurate localization of the target as well as the UAS and a 100% accurate target detection. Given the dynamic nature of the task and the outdoor environment, a 100% accurate target detection is nearly impossible [34,48]. For an example, we observed a large number of false detections when sudden lighting changes occurred due to the movement of rainy clouds. Moreover, image-based target positioning had errors due to factors such as lens distortion, imaging sensor resolution, GPS accuracy, barometric altimeter resolution and target height [49]. GPS sensors used in typical, low-cost UASs are also subject to drift [27]. This study has developed a framework to address these challenges with less computational requirements and validated the framework using two different test cases with different implementations of the target detection module. The navigation framework proposed in this paper can address these challenges independent from detection algorithm thus a reasonable accuracy from detection algorithms is adequate to perform the task.

When we consider one potential application of the framework such as search and rescue, in earlier works [33,34,35,36,37], UAS was limited in its use as only as a monitoring device. The framework proposed in this research further extends the capabilities of UASs to more active participation, such as conducting a detailed closer inspection or delivering emergency kits. In other studies on railway semaphore inspection [23] and pole inspection [24], the UAS was manually positioned at a workable distance, and researchers did not address the part of finding and approaching the target for inspection. The framework proposed in this research may be used in conjunction with those techniques to increase autonomy. This framework has opened the whole new avenue of research where UASs can be used for close inspection of ground targets for more informed decision-making or can be used to perform an action on the target.

The system presented in this paper uses a higher level decision-making approach OODA for control and has no operator involvement from the mission start to end, and can automatically re-plan the task in the absence of identified targets or if new targets appear. This ability meets level 4 autonomy, according to the autonomy level evaluation method of NASA [38]. However, the framework’s lack of the capability to avoid obstacles means it cannot perceive the environment widely as required by level 4 autonomy. This requirement needs to be addressed in the future to achieve a fully compatible level 4 autonomy.

The proposed framework has limitations. The voting-based scheme employed to remove false detections assumes that false detections are not always persistent. However, this might not be true for all target detection methods. Techniques used for target tracking assume errors and drift in sensors are gradual over time, which might fail when abrupt changes occur. The framework also assumes the targets are located in reasonably flat terrain. Although this assumption may be appropriate for agricultural land, further research is needed to address targets located in hilly or sloping terrain.

## 6. Conclusions

This paper proposed a framework for autonomous, multiple ground target-finding and action using a low-cost, multirotor unmanned aerial system. The framework can be effectively used in a variety of UAS applications with suitable detection algorithms in fully and supervised autonomous missions such as inspection, search and rescue and spot spraying in agriculture. Uncertainties and errors introduced by the onboard sensors and external disturbances such as wind pose significant challenges in real outdoor environments. A framework was proposed using the high-level decision-making approach of OODA, a series of techniques, and a modular software system to address these challenges.

Two different test cases were formulated to search and inspect multiple ground objects scattered on the ground. Each test case was repeated multiple times to show that the framework can overcome the errors and uncertainties and perform the task successfully. The detection algorithm is simple compared to the state-of-the-art algorithm used in precision agriculture or search and rescue work. There are two reasons for the simple algorithm: one is to demonstrate the capability of the framework with a high number of false detections, and the other one is the limited computation capacity of the Raspberry Pi used in our UAS.

Ongoing work focuses on integrating a sprayer system to a suitable UAS platform, identifying a test site, and obtaining approvals to test the system in paddy fields for brown planthopper control application.

A video of the flight tests can be found at https://www.youtube.com/watch?v=QVmHo_uUkfU.

## Figures and Tables

**Table 1:** Parameters used in the experiments.

Paramete	Value
Search height (*h_s_*)	40 m
Threshold rotation rate (*α*)	0.8 deg/s
Gating distance (*d*)	2 m
Camera frame rate	10 Hz
Camera resolution	640 × 480
Proportional gain (*K*)	2
Votes for a detection ($w_{d}$)	1
Votes for a non-detection ($w_{n}$)	−1
Removal cut-off (*G*)	−2
Valid target cut-off (*H*)	5

**Table 2:** Summary of results from flight tests.

Test No.	Number of Targets	Number of Targets Visited	Number of Targets Not Inspected (Action Failure)
1	6	6	1
2	6	5	0
3	6	5	0
4	6	6	1
5	6	6	1
